# Measuring quality and outcomes of research collaborations: An integrative review

**DOI:** 10.1017/cts.2019.402

**Published:** 2019-10-11

**Authors:** Beth B. Tigges, Doriane Miller, Katherine M. Dudding, Joyce E. Balls-Berry, Elaine A. Borawski, Gaurav Dave, Nathaniel S. Hafer, Kim S. Kimminau, Rhonda G. Kost, Kimberly Littlefield, Jackilen Shannon, Usha Menon, The Measures of Collaboration Workgroup of the Collaboration and Engagement Domain Task Force, National Center for Advancing Translational Sciences, National Institutes of Health

**Affiliations:** 1 University of New Mexico, College of Nursing, Albuquerque, NM, USA; 2 Department of Internal Medicine, University of Chicago Hospitals, Chicago, IL, USA; 3 Department of Family, Community and Health Systems, University of Arizona, College of Nursing, Tucson, AZ, USA; 4 Division of Epidemiology, Mayo Clinic, Rochester, MN, USA; 5 Department of Population and Quantitative Health Sciences, Case Western Reserve University School of Medicine, Cleveland, OH, USA; 6 Department of Medicine, University of North Carolina at Chapel Hill, Chapel Hill, NC, USA; 7 Center for Clinical and Translational Science, University of Massachusetts Medical School, Worcester, MA, USA; 8 University of Kansas Medical Center, Family Medicine and Community Health, Kansas City, KS, USA; 9 The Rockefeller University, Clinical Research Support Office, New York, NY, USA; 10 University of North Carolina-Greensboro, Office of Research and Engagement, Greensboro, NC, USA; 11 Oregon Health and Sciences University, OHSU-PSU School of Public Health, Portland, OR, USA; 12 University of South Florida College of Nursing, Tampa, FL, USA

**Keywords:** Team science, research collaboration, instrument development, psychometrics, outcome measure, research process measure, research evaluation, scientific collaboration, measures, teamwork

## Abstract

**Introduction:**

Although the science of team science is no longer a new field, the measurement of team science and its standardization remain in relatively early stages of development. To describe the current state of team science assessment, we conducted an integrative review of measures of research collaboration quality and outcomes.

**Methods:**

Collaboration measures were identified using both a literature review based on specific keywords and an environmental scan. Raters abstracted details about the measures using a standard tool. Measures related to collaborations with clinical care, education, and program delivery were excluded from this review.

**Results:**

We identified 44 measures of research collaboration quality, which included 35 measures with reliability and some form of statistical validity reported. Most scales focused on group dynamics. We identified 89 measures of research collaboration outcomes; 16 had reliability and 15 had a validity statistic. Outcome measures often only included simple counts of products; publications rarely defined how counts were delimited, obtained, or assessed for reliability. Most measures were tested in only one venue.

**Conclusions:**

Although models of collaboration have been developed, in general, strong, reliable, and valid measurements of such collaborations have not been conducted or accepted into practice. This limitation makes it difficult to compare the characteristics and impacts of research teams across studies or to identify the most important areas for intervention. To advance the science of team science, we provide recommendations regarding the development and psychometric testing of measures of collaboration quality and outcomes that can be replicated and broadly applied across studies.

## Introduction

Translating basic science discoveries into demonstrated improvements in public health requires a research team from diverse backgrounds [1–3]. The US National Institutes of Health National Center for Advancing Translational Sciences recognized this need by establishing a strategic goal to advance translational team science by fostering innovative partnerships and diverse collaborations [4]. In the health sciences, there is significant interest in translational research and moving more quickly from single-study efficacy trials to effective, generalizable interventions in health care practice. Foundational to this body of literature is the assumption that cross-disciplinary research teams speed the process of translational research [5].

Analyses of trends in scientific publications suggest that major advances in biological, physical, and social science are produced by research teams; that the work of these teams is cited more often than the work of individual researchers; and that, in the long term, the work has greater scientific impact [6–9]. In addition, cross-disciplinary diversity is assumed to lead to greater innovation [10]. These observations have become the cornerstone of the translational science movement in the health sciences.

Implementing team science can be challenging. Multiple authors have noted that working in collaboration can be more expensive and labor intensive than working alone [11,12]. Noted trade-offs include added time and effort to communicate with diverse collaborators, conflicts arising from different goals and assumptions, and increased start-up time with its resulting delay in productivity [13–17]. These opportunity costs may be acceptable if the outcomes of research collaborations can accelerate knowledge or answer the complex health questions faced by today’s society.

To test the assumption that research collaboration leads to greater productivity, we need to accurately measure the characteristics of research teams and their outcomes and be able to compare results across teams [6,12,15,18–27]. Although different measures have so far shown that collaborations are beneficial, operational definitions of variables that may influence conclusions (construct validity) are varied, complicating interpretation of results. Despite some exceptions [12,19,23,28], there is a lack of attention to the development and psychometric testing of reliable and valid measures of collaboration. As an initial step, it would be useful to have an overview of the current state of the science in the measurement of research collaborations. In this article, we report the results of an integrative review of the literature, looking for reliable and valid measures that describe the quality and outcomes of research collaborations.

## Materials and Methods

We conducted two reviews. The first focused on measures of collaboration quality, defined as measures of interactions or processes of the team during the collaboration. The second review focused on outcomes of the collaboration (e.g., publications, citations). We used an integrative review approach. An integrative review is a specific type of review that applies a comprehensive methodology involving a combination of different approaches to summarize past research related to a particular topic, including both experimental and non-experimental studies, and reach conclusions [29,30].

Our research team brainstormed keyword combinations and, based on expert opinion, agreed on final sets of keywords that were comprehensive enough to cover the topics fully but not so broad as to include non-relevant literature. For the review of collaboration quality, these keywords were “measure/measurement” combined with the following terms: community engagement, community engaged research, collaboration, community academic partnership, team science, regulatory collaboration, industry collaboration, public–private partnership (focus on research). For the review associated with collaboration outcomes, the word “outcomes” was added to the above search terms. Our intention was to include all types of research collaborations, including partnerships between academic and other community, governmental, and industry partners. The following keywords were considered, tested in preliminary searches, and eliminated by group consensus as being too broad for our purpose: consortium collaboration, public health and medicine collaboration, patient advocacy group collaboration, and coalition. Measures of collaboration related to clinical care, education, and program delivery collaborations were excluded from this review.

**Fig. 1.**
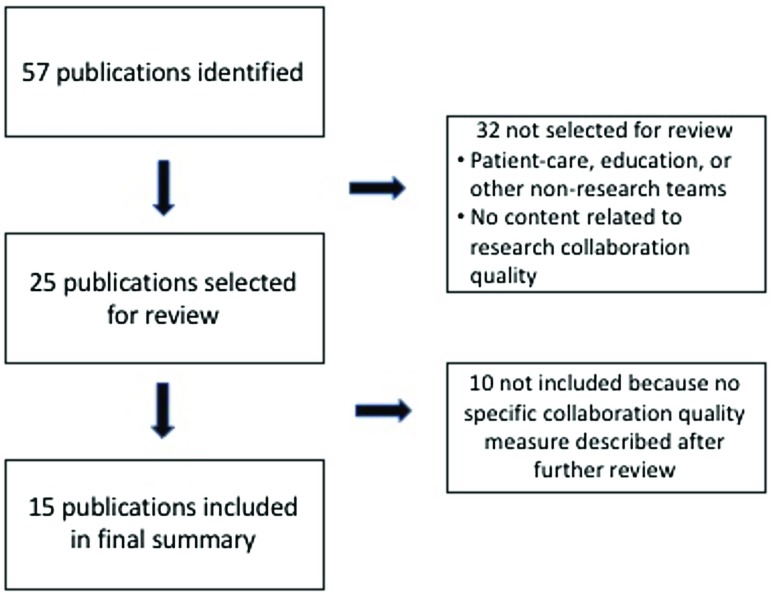
Flow diagram of publications included in the final collaboration quality review.

**Fig. 2.**
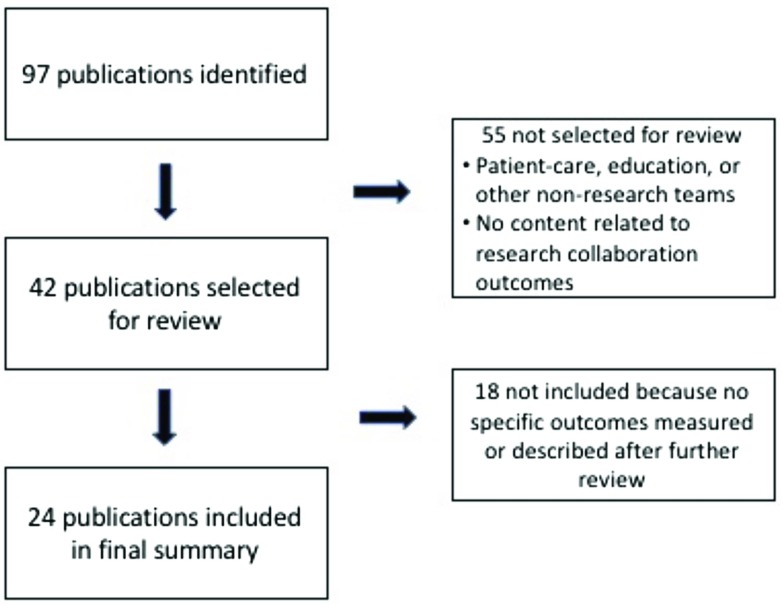
Flow diagram of publications included in the final collaboration outcomes review.

Quality and outcome measures were identified using both a literature review and an environmental scan. We conducted searches using the standard databases PubMed, the Comprehensive Index to Nursing and Allied Health Literature, and PsychInfo, as well as searched EMBASE, Google Scholar, Scopus, and websites recommended by members of the research team. After duplicates and articles that were not focused on a specific scale or measure of research collaboration were eliminated, team members reviewed a final list of 25 publications for the measures of collaboration quality, including 4 articles describing social network analyses, and 42 publications for measures of collaboration outcome. All publications were published prior to 2017. Figs. 1 and 2 provide flow diagrams of how articles were selected to be included in both reviews.

At least two members of the research team reviewed each article using a standard data abstraction form that included the name of the measure/outcome; construct being measured; sample; and details about the measure, including operational definition, number of items, response options, reliability, validity, and other evidence for supporting its use. Reviewers were also asked to make a judgment as to whether the article included a measure of the collaboration quality (or outcomes or products) of the scientific/research collaborations; both reviews had a rater agreement of 99%. Differences in reviews were resolved through consensus after discussions with a third reviewer.

## Results

### Quality Measures

We identified 44 measures of research collaboration quality from the 15 publications included in the final summary analyses (see Fig. 1). The specifics of each measure are detailed in Table 1. Three articles were not included in Table 1 because they all used social network analysis [31–33]. Four articles covered 80% of the measures identified [12,19,23,34].

**Table 1.** Measures of research collaboration quality

Author	Instrument name	Construct being measured	Sample/ population	Number of items	Response options	Reliability	Validity
Bietz et al. [36]	Collaboration Success Wizard	Past, present, or future perceptions of five factors: nature of work; common ground; collaboration readiness; management, planning and decision making; and technology readiness	177 university faculty and staff from 12 projects	44	Varied	NR	NR
Greene et al. [37]	CRN Participant Survey	Five domains: extent of collaboration and quality of communication; performance of projects and infrastructure; data quality; scientific productivity; and impact on member organizations	Investigators and project staff from the HMO Cancer Research Network over a 5-year period	All items not provided; questions modified annually based on feedback and new project needs	Several 5-point Likert scales ranging from “strongly agree” to “strongly disagree” or from “very effective” to “very ineffective, including “can’t evaluate”; also, open-ended items to collect qualitative input		
Hall et al. [12]	Research Orientation Scale	Unidisciplinary, multidisciplinary, or inter/transdisciplinary proclivity of values and attitudes toward research	56 investigators and staff from four NCI TREC centers	10	5-point Likert scale ranging from “strongly agree” to “unsure” to “strongly disagree”	Cronbach’s alpha = 0.74	Construct validity: Exploratory and confirmatory factor analyses supporting three factors. Convergent validity: Higher unidisciplinary orientation inversely correlated with cross-disciplinary collaborative activities and multidisciplinary and inter/transdisciplinary research orientation. Higher multidisciplinary orientation correlated with more collaborators, more cross-disciplinary collaborative activities. Similar findings for inter/transdisciplinary research orientation
	Completing Deliverables Scale	Investigators’ expectations for their projects’ meeting projected year-1 deliverables		One item for each project	5-point Likert scale ranging from “highly unlikely” to “highly likely”; each project was rated separately	NA	Convergent validity: Inverse correlation between duration of involvement in transdisciplinary projects at their center and researchers’ confidence in meeting year 1 deliverables
Hall et al. [12]^a^	History of Collaboration (other individual investigators)	Number of individuals collaborated with and length of time for each individual	56 investigators and staff from four NCI TREC centers	2	Count of collaborators; number of years of collaboration for each collaborator		Convergent validity: Number of collaborators correlated with more center-related collaborative activities, number of years working in inter/transdisciplinary centers, number of years working with inter/transdisciplinary projects, higher multidisciplinary, and inter/transdisciplinary research orientation
	History of Collaboration (other centers or projects)	Number of years in inter/transdisciplinary center and projects		4	Counts of number of centers and of years involved with center; counts of number of projects and number of years involved with projects		Convergent validity: More years involved in inter/transdisciplinary centers correlated with number of collaborators and inversely correlated with confidence in completion of 1-year deliverables. More years involved in inter/transdisciplinary projects correlated with number of collaborators and inversely correlated with confidence in completion of 1-year deliverables
	Satisfaction with Collaboration	Satisfaction with each individual collaborator		1	5-point Likert scale ranging from “not at all satisfied” to “neutral” to “completely satisfied”		Convergent validity: More satisfaction with individual collaborations correlated with more perceived institutional resources supporting collaboration, more positive impressions, higher ratings of interpersonal collaborations, higher perception of collaborative productivity
	Cross-Disciplinary Collaboration-Activities Scale	Frequency of engagement in collaborative activities outside of his/her primary field		6	7-point Likert scale ranging from “never” to “weekly”	Cronbach’s alpha = 0.81	Convergent validity: higher frequency of cross-disciplinary collaborative activities correlated with stronger multidisciplinary and interdisciplinary/transdisciplinary research orientation
	TREC-related Collaborative Activities Scale	Frequency of engagement with center-specific activities		3		Cronbach’s alpha = 0.74	
	Institutional Resources Scale	Availability and quality of institutional resources for conducting collaborative research project		8	5-point Likert scale ranging from “very poor” to “excellent”	Cronbach’s alpha = 0.87	Convergent validity: The better the perceived resources, the more positive researchers’ perception of center, more satisfied with previous collaborators, more positively rated collaborative productivity and interpersonal collaboration
	Semantic-Differential/ Impressions Scale	Investigators’ impressions of their research center		21 adjective pairs	7-point continuum on which respondents rate their impressions on adjective pairs (e.g., conflict-harmonious, not supportive-supportive, fragmented-integrated)	Cronbach’s alpha = 0.98	Convergent validity: Better impressions were correlated with more collaboration satisfaction, more confidence in completion of deliverables, more institutional resources for collaboration, better interpersonal collaboration
	Interpersonal-Collaboration Scale	Interpersonal collaborative process at their center		8	5-point Likert scale with response options ranging from either “very poor” to “excellent” or “strongly agree” to “strongly disagree” with central “neither agree nor disagree”	Cronbach’s alpha = 0.92	Convergent validity: The better the interpersonal collaboration, the more collaboration satisfaction, the more confidence in completion of deliverables, and the more perceived institutional resources for collaboration
Hall et al. [12]^b^	Written Products Protocol	The integrative (transdisciplinary) aspects of written research protocols, disciplines represented, levels of analysis, type of cross-disciplinary integration	21 center developmental project proposals from four NCI TREC centers	37 item protocol used to evaluate proposals	Items describing proposal with various response formats; one item – rate whether “unidisciplinary,” “multidisciplinary,” “interdisciplinary,” or “transdisciplinary” proposal, two items regarding transdisciplinary integration and scope of proposal using 10-point Likert scale ranging from “none” to “substantial”	Inter-rater reliabilities based on Pearson’s correlations from 0.24 to 0.69; highest reliability for rating experimental types (0.69), number of analytic levels (0.59), disciplines (0.59) and scope (0.52). Lower reliability in attempts to name the cross-disciplinary integration in the proposal	Convergent validity: Higher number of disciplines in proposal, the broader its integrative score, larger its number of analytic levels. The higher the type of disciplinarity, the broader its overall scope
Huang [34]	Trust	Team trust	290 members of 60 technology research and development teams from the Industrial Technology Research Institute in Taiwan	7	5-point Likert scale ranging from “strongly disagree” to “strongly agree”	Cronbach’s alpha = 0.87; ICC = 0.46 (*p* < 0.001)	Face and content validity. Construct validity: Common method variance analysis and confirmatory factor analysis using partial least squares latent structural modeling. Convergent validity with other constructs with composite reliability > 0.6 and average variance extracted at least 0.5. Discriminant validity with square root of average variance for construct greater than levels of correlations involving construct
	Transactive Memory System	Team transactive memory system		7		Cronbach’s alpha = 0.84; ICC = 0.42 (*p* < 0.001)	
	Knowledge Sharing	Team knowledge sharing		4		Cronbach’s alpha = 0.79; ICC = 0.55 (*p* < 0.001)	
	Group cohesiveness	Group cohesiveness (team network ties and collective mind)		5		Cronbach’s alpha = 0.78; ICC = 0.42 (*p* < 0.001)	
	Team Performance	Team performance		4		Cronbach’s alpha = 0.82; ICC = 0.53 (*p* < 0.001)	
Lee and Bozeman [38]	Collaboration Strategies	Collaboration strategies or motives for collaboration	443 science faculty affiliated with NSF or DOE research centers at US universities	13	4-point Likert scale ranging from “very important” to “not important”	Cronbach’s alpha for subscales: Taskmaster (2 items) = 0.60; Nationalist (2 items) = 0.57; Mentor (2 items) = 0.57; Follower (3 items) = 0.42; Buddy (3 items) = 0.32; Tactition only one item. No overall Cronbach’s alpha reported	Construct validity: Exploratory factor analysis with varimax rotation supporting six factors: Taskmaster, Nationalist, Mentor, Follower, Buddy, and Tactition. Convergent validity: Nationalist and Mentor collaboration strategies/motives significantly associated with number of collaborators during past 12 months. Only Tactition strategy/motive significantly associated with journal publication productivity as measured by a normal count and a fractional count of publications during the 3 years post survey
	Collaboration	Number of collaborators		1	Count of number of persons, by category, with whom they engaged in research collaborations within the past 12 months. Categories were male university faculty, male graduate students, male researchers who are not university faculty or students, female university faculty, female graduate students, and female researchers who are not university faculty or students	NA	Convergent validity: Zero order correlations between number of collaborators and journal publication productivity, both normal count and fractional count. Two-stage least squares regression results, including other moderating variables, demonstrated a continued significant relationship between number of collaborators and normal publication count
Mallinson et al. [39]	MATRICx	Motivators and threats to collaboration readiness	125 faculty, students, researchers	48 (31 threat and 17 motivator items)	4-point Likert scale: 4 = describes me/my experience exactly; 3 = describes me/my experience quite well; 2 = somewhat describes me/my experience; 1 = does not describe me/my experience at all	Rasch analysis: Person separation reliability for threat items = 0.92; motivator items (experienced participants) = 0.94; motivator items (inexperienced participants) = 0.85; all items = 0.67	Construct validity: Rasch analysis and principal components analysis
Mâsse et al. [19]^c^	Transdisciplinary Integration Scale	Attitudes about transdisciplinary research	216 research faculty, staff, trainees from NCI TTURC	15	5-point Likert scale ranging from “strongly agree” to “unsure” to “strongly disagree”	Cronbach’s alpha = 0.89	Construct validity: Confirmatory factor analysis. Convergent validity: Correlations with center outcomes
	Satisfaction with Collaboration	Satisfaction with collaboration within a center		8	5-point Likert scale ranging from “inadequate” to “excellent”	Cronbach’s alpha = 0.91	Construct validity: Confirmatory factor analysis. Convergent validity: Satisfaction with collaborations within a center correlated with center outcomes related to methods, science and models, and improved interventions
	Impact of Collaboration	Impact of collaboration within a center		5	3 items (meeting, products, overall productivity)-5-point Likert scale ranging from “inadequate” to “excellent”; remaining two items (research productivity, quality research)-5-point Likert scale ranging from “strongly disagree” to “not sure” to “strongly agree”	Cronbach’s alpha = 0.87	Construct validity: Confirmatory factor analysis. Convergent validity: Impact of collaborations within a center correlated with center outcomes related to methods, science and models, and improved interventions
	Trust and Respect	Trust and respect with collaborations		4	5-point Likert scale ranging from “strongly disagree” to “not sure” to “strongly agree”	Cronbach’s alpha = 0.75	Construct validity: Confirmatory factor analysis. Convergent validity: Trust and respect with collaborations within a center correlated with center outcomes related to methods, science and models, and improved interventions
Mazumdar et al. [40]	No formal name	Team scientists’ activities related to grant design, grant implementation, grant analysis, manuscript reporting, teaching, and service	Proposed for academic faculty	6	3 possible ratings for each item: major, moderate, or minor; Supported by qualitative comment by reviewer	NR	
Misra et al. [28]	Transdisciplinary Orientation Scale	Values, attitudes, beliefs, conceptual skills, knowledge and behavioral repertoires that predispose an individual to collaborating effectively in cross-disciplinary scientific teams	150 researchers and academics from the liberal arts, social sciences, natural sciences, and engineering	12	5-point Likert scale ranging from “strongly agree” to “strongly disagree”; middle three response options not anchored	Overall Cronbach’s alpha = 0.93 (values, attitudes, beliefs 6-item subscale alpha = 0.87; conceptual skills and behaviors 6-item subscale alpha = 0.88); second sample Cronbach’s alpha = 0.92	Construct validity: Confirmatory factor analysis. Convergent validity: Critical ratio of regression weights statistically significant. Discriminant validity: Covariance between two factors and correlation high but suggests discriminant validity. In multiple regression analyses, higher transdisciplinary orientation associated with production of more interdisciplinary scientific papers, more experience in participating in cross-disciplinary team science, and independent ratings of the potential society impact of the research reported in the scholar’s article
Oetzel et al. [23]^d^	Bridging Social Capital	Academic and community partners have the skills and cultural knowledge to interact effectively Domain: Structural/individual dynamics	138 PIs/PDs and 312 academic or community partners from 294 CBPR projects with US federal funding in 2009	3	5-point Likert scale: 1=not at all; 2=very little; 3=somewhat; 4=mostly; 5=to a great extent	Cronbach’s alpha = 0.69	Construct validity: Confirmatory factor analysis. Convergent validity: Positive and moderate correlations with other structural/individual dynamics scales; and correlations with multiple outcomes
	Alignment with CBPR Principles: Partner Focus	Develops individual partner capacity and equitable partnerships in all phases of the research Domain: Structural/individual dynamics		4		Cronbach’s alpha = 0.82	
	Alignment with CBPR Principles: Community Focus	Builds on resources and strengths of community for the well-being of community Domain: Structural/individual dynamics		4		Cronbach’s alpha = 0.85	
	Partner values	Shared understanding of project mission, priorities, strategies Domain: Structural/individual dynamics		4	5-point Likert scale: 1 = strongly disagree; 2 = disagree; 3 = neither agree nor disagree; 4 = agree; 5 = strongly agree	Cronbach’s alpha = 0.89	
	Research Tasks and Communication: Background Research	Community partners’ level of involvement in the background research Domain: Relational dynamics		5	1 = community partners DID NOT/DO NOT participate in this activity; 2 = community partners were/are CONSULTED on this activity; 3 = community partners were/are ACTIVELY ENGAGED in this activity; 4 = not at this stage of research; 5 = does not apply	Cronbach’s alpha = 0.81	Construct validity: Confirmatory factor analysis. Convergent validity: Positive and moderate correlations with other relational dynamics scales; and correlations with multiple outcomes
	Research Tasks and Communication: Data Collection	Community partners’ level of involvement in the data collection Domain: Relational dynamics		4		Cronbach’s alpha = 0.69	
	Research Tasks and Communication: Analysis and Dissemination	Community partners’ level of involvement in data analysis and dissemination of findings Domain: Relational dynamics		3		Cronbach’s alpha = 0.82	
	Dialogue and Mutual Learning: Participation	Degree to which all partners participate in the process Domain: Relational dynamics		3	5-point Likert scale: 1 = strongly disagree; 2 = disagree; 3 = neither agree nor disagree; 4 = agree; 5 = strongly agree	Cronbach’s alpha = 0.78	
	Dialogue and Mutual Learning: Cooperation	Degree to which partners cooperate to resolve disagreements Domain: Relational dynamics		3		Cronbach’s alpha = 0.83	
	Dialogue and Mutual Learning: Respect	Degree to which partners convey respect to each other Domain: Relational dynamics		3		Cronbach’s alpha = 0.83	Construct validity: Confirmatory factor analysis. Convergent and discriminant validity: Mixed results
	Trust	Degree of current trust within partnership Domain: Relational dynamics		4		Cronbach’s alpha = 0.86	Construct validity: Confirmatory factor analysis. Convergent validity: Positive and moderate correlations with other relational dynamics scales; and correlations with multiple outcomes
	Influence and Power Dynamics	Degree of voice and influence in the decision-making Domain: Relational dynamics		3		Cronbach’s alpha = 0.58	
	Participatory Decision-Making	Degree to which decisions are made in a participatory manner Domain: Relational dynamics		4	5-point Likert scale: 1 = never; 2 = rarely; 3 = sometimes; 4 = often; 5 = always	Cronbach’s alpha = 0.83	
	Leadership	Overall effectiveness of project’s leadership Domain: Relational dynamics		10	5-point Likert scale: 1 = very ineffective; 2 = ineffective; 3 = somewhat effective; 4 = effective; 5 = very effective	Cronbach’s alpha = 0.94	
	Resource Management	Effective use of financial and in-kind resources Domain: Relational dynamics		3	5-point Likert scale: 1 = makes poor use; 2 = makes fair use; 3 = makes average use; 4 = makes good use; 5 = makes excellent use	Cronbach’s alpha = 0.86	
Okamoto et al. [41]	Conflict	Task conflict	167 center directors, research core PIs, individual project PIs, key research personnel from 10 US National Institutes of Health Centers for Population Health and Health Disparities	6	5-point Likert scale from “not at all” to “to a very large extent”	Cronbach’s alpha = 0.82	Discriminant validity: Task conflict not associated with any of the three network measures
Wooten et al. [27]	Team Evaluation Model Matrix	Functioning of multidisciplinary translational teams within a two-by-two matrix based on assessment of two dimensions: Team maturation/development and; research and scientific progress	11 Multidisciplinary translational research teams within one US National Institutes of Health, National Center for Advancing Translational Science, Clinical and Translational Science Award center	2	Expert panel members rated each team on each two dimensions (Maturation/development; and Research/scientific) on a scale with 0 = not present; 1 = low; 2 = medium; 3 = high. On each dimension could have a final score of 0–12 (0–3 scores for each of four criteria under each dimension). After initial scoring by individual panel members, total expert panel discuss to reach final consensus on each team’s scores on each of the two dimensions. Initial ratings based on expert panel members’ review of team logic model, measurement plan, and all assessment data (including survey data)	NR	NR

NR, not reported; CRN, Cancer Research Network; NCI, US National Cancer Institute; TREC, Transdisciplinary Research on Energetics and Cancer; ICC, intraclass correlation coefficients; NSF, US National Science Foundation; DOE, US Department of Energy; MATRICx, Motivation Assessment for Team Readiness, Integration and Collaboration; NA, not applicable; TTURC, Transdisciplinary Tobacco Use Research Center; PI, principal investigator; PD, project director; CBPR, community-based participatory research.

a Details obtained by cross-referencing article (TREC Baseline survey) from https://www.teamsciencetoolkit.cancer.gov/Public/TSResourceMeasure.aspx?tid=2%26rid=36 [42].

b Detail obtained by cross-referencing article (NCI TREC Written Products Protocol 2006-09-27) from https://www.teamsciencetoolkit.cancer.gov/Public/TSResourceMeasure.aspx?tid=2%26rid=646 [43].

c Details obtained by cross-referencing article (TTURC Researcher Survey 2002) from https://cctst.uc.edu/sites/default/files/cis/survey-TTURC_research.pdf [44].

d Original instrument shown at http://cpr.unm.edu/research-projects/cbpr-project/index.html--scroll to 2. Quantitative Measures – “Key Informant” and “Community Engagement” survey instruments. Developmental work on measures from Oetzel et al. (2015) continues in an NIH NINR R01 (Wallerstein [PI] 2015-2020 “Engage for Equity” Study; see http://cpr.unm.edu/research-projects/cbpr-project/cbpr-e2.html).

The number of items per measure ranged from 1 to 48, with 77% having less than 10 items per measure. A few articles reported on measures that covered several domains. As shown in Table 1, we have included each domain measure separately if it was reported as an independent scale with its own individual psychometric properties.

Reliability was reported for 35 measures, not reported for four measures, and not applicable for five measures (single-item, self-reported frequency counts, or qualitative responses). Reliability measures were most frequently Cronbach’s alphas for internal consistency reliability, but also included intraclass correlation coefficients, inter-rater correlations, and, when Rasch analysis was used, person separation reliability. Test–retest reliability was never reported. Cronbach’s alpha statistics were >0.70 for 86% of the measures using that metric. Some form of validity was reported on 40 measures and typically included exploratory (*n* = 8) and/or confirmatory factor analysis (*n* = 26). Convergent or discriminant validity was evident for 38 measures but was based on study results, as interpreted by our reviewers, rather than identified by the authors as a labeled multitrait–multimethod matrix analysis of construct validity. Twelve measures had convergent or discriminant validity only, without any further exploration of validity. Face validity and content validity were reported for five measures, along with other analyses of validity.

### Outcome Measures

We identified 89 outcome measures from the 24 publications included in the final summary analyses (see Fig. 2). Characteristics of each measure are detailed in Table 2. Three publications included over 44 (49%) of the measures identified [17,23,35]. However, only two of those [17,23] included measures tested in actual studies; the remaining article [35] included only recommendations for specific measures.

**Table 2.** Measures of research collaboration outcomes

Author	Outcome	Sample/ population	Detail about outcome measurement	If scale: number of Items	If scale: response options	Reliability	Validity
***Counts or numerical representations of products***							
Ameredes et al. [45]	Number of extramural grants submitted and received	110 trainees involved in multidisciplinary translational teams at one NIH CTSA institution	Not specified how data for counts obtained	NA	NA	NR	NR
	Number of publications over 3-year period		Not specified how data for counts obtained				
Cummings et al. [13]	Number of publications in final NSF reports over 4- to 9-year period	549 research groups funded by NSF from 2000 to 2004	Count of publications for each research group listed in final report to NSF or, if no final report, last annual report, between 2000 and 2009. Included archival conference proceedings, journal articles, book chapters, public reports on project. Each group’s publications counted only once, regardless of number of co-authors				
	Number of cumulative publications for research group pre/post NSF funding listed in Google Scholar		Count of publications for each research group extracted through Google Scholar search engine divided into pre-NSF funding and post-NSF funding (through 2009). Each group’s publications counted only once, regardless of number of co-authors			Reliability evaluated for 10% of sample using raters recruited via Amazon’s Mechanical Turk. 5 raters compared each extracted publication with corresponding author’s Web page or resume publication listings. Publication counted if 4 of 5 raters agreed. Rater agreement was 94%	
	Number of cumulative publications for research group pre/post NSF funding listed in Thomas Reuters [formerly ISI] Web of Science and Social Science database		Count of publications for each research group extracted through Thomas Reuters (formerly ISI) Web of Science and Social Science database divided into pre-NSF funding and post-NSF funding (through 2009). Each group’s publications counted only once, regardless of number of co-authors				
	Number of cumulative citations for research group publications pre/post NSF funding listed in Thomas Reuters (formerly ISI) Web of Science and Social Science database		Count of citations of each unique publication for each research group extracted through Thomas Reuters (formerly ISI) Web of Science and Social Science database divided into pre-NSF funding and post-NSF funding (through 2009). Each group’s publications counted only once, regardless of number of co-authors				
Hughes et al. [33]	Number of co-authored publications over 4-year period	Active investigators at one NIH CTSA site	Count of co-authored publications between 2006 and 2009 involving two or more investigators. Used Ruby scripts to automatically harvest publication information from the US National Center for Biotechnology Information’s PubMed database			NR	
	Number of co-authored grant proposals over 4-year period		Count of co-authored grant proposals submitted between 2006 and 2009 involving two or more investigators (institutional data and additional data from NIH RePORT)				
Lee and Bozeman [38]	Number of peer-reviewed journal papers (as a measure of individual productivity)	443 science faculty affiliated with NSF or DOE research centers at US universities	Count of peer-reviewed journal papers from 2001-2003 obtained from SCI-Expanded through the ISI Web of Science. Authors identified by matching name, department, and institution found on respondent’s CV				
	Fractional count of co-authored peer-reviewed journal papers (divided by number of co-authors) (as a measure of individual productivity)		Count of co-authored peer-reviewed journal papers divided by number of co-authors from 2001 to 2003 obtained from SCI-Expanded through the ISI Web of Science. Authors identified by matching name, department, and institution found on respondent’s CV				
Lee [46]	Number of invention disclosures	427 university faculty – science and engineers from research-intensive universities on the NSF list of top 100 research universities	Self-reported number on one-time anonymous survey				
	Number of patents obtained		Self-reported number on one-time anonymous survey				
	Number of patents pending		Self-reported number on one-time anonymous survey				
Lööf and Broström [47]	Income from new or improved products introduced during 1998-2000 as a proportion of sales income in year 2000	2071 manufacturing and business firms in Sweden that have collaborations with universities – data from Community Innovation Survey III in Sweden 1998-2000	Survey data reported by firms; income from new or improved products introduced during 1998-2000 as a proportion of sales income in year 2000				
	Number of patent applications by industry partners in 2000		Survey data reported by firms; count of patent applications by industry partners in 2000				
Luke et al. [48]	Number of grant submissions over 4-year period	1272 research members of one institutional NIH CTSA center	Counts of new extramural submissions over 4-year period as maintained in university database, including federal, state, local, and foundation grants, contracts, programs, and sub-agreements, excluding renewals, resubmissions, etc.				
	Number of publications over 4-year period		Counts of publications over 5-year period based on bibliometric data obtained from Elsevier *Scopus*				
Mâsse et al. [19]	Number of submitted and published articles and abstracts	216 research faculty, staff, and trainees from NCI TTURC	Counts of submitted and published articles and abstracts (to date) reported in written survey by participants (research faculty, staff, and trainees) in Year 3 of center				
Petersen [49]	Normalized number of publications per year	473 pairs of research collaborators who published in Thompson Reuters Web of Knowledge publications (spanning 15,000 career years, 94,000 publications, and 166,000 collaborators)	Publication counts aggregated over relevant time periods, normalized by the baseline average calculated over the period of analysis				
	Normalized number of citations per year		Citation count in a given census year converted to a normalized *z* score (to correct for older publications that have more time to accrue citations than newer publications)				
Philbin [35]	Number of publications and conference proceedings (as indicator of technology knowledge sharing and improvement)	None – measure proposed based on lit review and interviews with 32 university and industry representatives involved in research collaborations	Counts of publications in scientific journals and peer-reviewed conference proceedings (no suggestion regarding what data source would be used to ascertain counts)			NA	NA
	Quality of research publication as measured by a citation index (as indicator of technology knowledge sharing and improvement)		Citation index value for each publication (exact citation index not specified)				
	Number of students associated with collaboration (as indicator of technology knowledge sharing and improvement		Count of number of students, including postgraduate masters and PhD levels, associated with the collaboration				
	Financial value of projects according to sponsor and sector (as indicator of project and business knowledge sharing and improvement)		US dollar calculation of financial value of projects according to sponsor and sector, including measures for growth and decline and market share (no calculation details provided)				
	Third-party recognition of collaboration results (e.g., awards) (as indicator of technology sustainability of collaboration)		Level of third-party recognition of collaboration results (e.g., number of awards) (no specifics regarding measurement other than count of awards)				
	Number of university students recruited as new staff into the company (as indicator of social sustainability of collaboration)		Number of students from the university recruited as new staff into the company (no specifics provided)				
	Completion of project milestones or deliverables (as indicator of projects and business knowledge sharing and improvement)		Description of completion of project milestones or deliverables that were achieved according to time, cost, and quality requirements (no specifics provided)				
	Number of staff exchanges and student placements (as indictor of social knowledge sharing and improvement)		Counts of staff exchanges and student placements (no specifics provided)				
	Attendance at key events (as indicator of social knowledge sharing and improvement)		Percentage attendance at key events, such as customer and milestone reviews and invited lectures (no specifics as to what the denominator would be)				
	Value of follow-on work and spin-off projects that have arisen as a consequence of initial funding (as indicator of project and business sustainability of collaboration)		Value of follow-on work and “spin-off” projects that have arisen as a consequence of initial funding (no specifics of how value would be quantified)				
	Value of intellectual property including patents and license agreements arising from the collaboration (as indicator of project and business sustainability of collaboration)		Value of intellectual property including patents and license agreements arising from the collaboration (no specifics regarding how value is quantified)				
	Long-term return on investment accrued from research investment (as indicator of project and business sustainability of collaboration)		Level of long-term return on investment accrued from research investment (no specifics provided)				
	Efficiency of contract management (as indicator of projects and business knowledge sharing and improvement)		Measure of the efficiency of contract management (e.g., submission of invoices) (no specifics provided)				
	Extent of adoption of research results in new products and services developed by the company (as indicator of technology sustainability of collaboration)		Extent of adoption of research results in new products and services by the company (no specifics provided)				
Stvilia et al. [50]	Number of publications	89 scientific teams conducting experiments at the NHMFL between 2005 and 2008	Counts of publication from a list of publications between 2005 and 2009 downloaded from the NHMFL website			NR	NR
Trochim et al. [17]	Number of citations: total, self-adjusted, and expected	216 research faculty, staff, and trainees from NCI TTURC	Bibliometric analysis of publications resulting from TTURC research and citing TTURC grant. Analysis produces both total, self- adjusted, and expected citation counts				
Wang and Hicks [51]	Average number of citations for new or repeated co-author teams	43,996 publications data from 1310 US scientists funded by NSF	Average number of forward citations (over 5-year period post publication) per paper for new and repeated (within last 3 years) co-author team papers based on lifetime publication data from Thomas Reuters Web of Science				
Wuchty et al. [8]	Number of citations for each paper/patent	19.9 million papers in the ISI Web of Science database and 2.1 million patents (all US patents registered since 1975)	Count of all research articles in ISI Web of Science database published since 1944; count of all US registered patents since 1975				
***Quality indicators of counted products***							
Lee et al. [52]	Novelty of research	1493 research-active faculty in science, engineering, and social sciences with publications included in Thomson Reuters Web of Science with at least two authors from the same institution on the paper	Formula cited in Lee et al. (2014) paper uses two steps: (1) calculate the commonness of co-cited journal pairs for the whole Web of Science database; (2) calculate the novelty of papers based on their references for the sampled papers (and taking only the 10th percentile)	NA	NA	NR	NR
	Impact of research based on citation percentiles		High impact defined as being in the top 1% of most cited papers in that Web of Science field in that year				
Trochim et al. [17]	Journal impact factor for each publication	Research faculty, staff, and trainees at NCI TTURC	Bibliometric analysis of journal impact factor for each publication resulting from TTURC research and citing TTURC grant; defined as average number of citations of a journal of all articles published in previous 2 years				
	Journal performance indicator for each publication		Bibliometric analysis of journal performance indicator for each publication resulting from TTURC research and citing TTURC grant; defined as average number of publications to date for all publications in a journal in a particular year				
	Field performance indicator for each publication		Bibliometric analysis of field performance indicator for each publication resulting from TTURC research and citing TTURC grant; defined as journal performance indicator for all journals in a field				
	5-Year Journal IF for each publication		Bibliometric analysis of 5 Year journal IF for each publication resulting from TTURC research and citing TTURC grant; defined as average number of citations to publications over a 5-year period				
Wuchty et al. [8]	RTI with and without self-citations	19.9 million papers in the ISI Web of Science database and 2.1 million patents (all US patents registered since 1975)	RTI = Mean number of citations received by team-authored work divided by the mean number of citations received by solo-authored work (>1 = team produced more highly cited papers than sole authors; <1 = vice versa; if = 1, no difference between sole and team authors)				
***Self-reported perceptions of outcomes***							
Ameredes et al. [45]	Perceived competency (confidence) in NIH CTSA recommended translational research competencies	32 early career scholars at one NIH CTSA institution	Each of 99 items (reflecting 15 competencies) rated in written survey by participants; final score used in analysis was an average of items for each competency sub-scale	99	6-point scale ranging from 0 to 5; specific anchors not provided; higher scores indicated confidence	NR	Construct validity: Principal components analysis
Greene et al. [37]	Perceived impact. Unclear because only have sample items and scales not constructed. Six sample questions appear to measure perceived impact on health plan, research organization, and individual	Investigators and project staff from the HMO Cancer Research Network over a 5-year period	Measured using structured questions with Likert-type responses included in annual survey sent to all consortium sites/members	Six sample questions only	4-point Likert scale ranging from “agree” to “disagree” (with a “can’t evaluate” option)		NR
Hager et al. [53]	Perceived research self-efficacy (skill)	Six interprofessional faculty fellows (dentists, pharmacists, physicians)	Research self-efficacy scale developed by Bieschke, Bishop and Garcia [54]	Not specified	100-point response scale; no anchors provided		
Hall et al. [12]	Investigators’ perceptions of center as a whole, as well as how they feel as a member of center in first year of center	56 Investigators and staff from four NCI TREC Centers	Semantic-differential/impression Scale: Ratings on a 7-point continuum on word/phrase pairs such as conflicted – harmonious; not supportive –supportive; scientifically fragmented – scientifically integrated	Not specified	7-point continuum; no anchors provided	Cronbach’s alpha = 0.98	
	Completing Deliverables Scale: Investigators’ expectations for their projects’ meeting projected year 1 deliverables		Not specified how final score computed	One item for each project	5-point Likert scale ranging from “highly unlikely” to “highly likely”; each project rated separately	NA	Convergent validity: Inverse correlation between duration of involvement in transdisciplinary projects at their center and researchers’ confidence in meeting year 1 deliverables
Hall et al. [12]^a^	Collaborative Productivity Scale: Perception of collaborative productivity within center, including productivity of scientific meetings, centers’ overall productivity		Rate the collaboration within your center: Productivity of collaborative meetings, overall productivity of center (rated on 5-point scale “very poor” to “excellent”); in general, collaboration has improved your research productivity (5-point scale from “strongly disagree” to “strongly agree”). Unclear whether three items were summed, summed and averaged, or if some other calculation was used to determine final scale value	Unclear; appears to be three items	5-point Likert scale ranging from “very poor” to “excellent”. Also asked to respond to a statement about collaboration and research productivity, rating on a 5-point scale from “strongly disagree” to “strongly agree” with central “neither” response option	Cronbach’s alpha = 0.95	Convergent validity: the better the perceived collaborative productivity, the better the collaboration satisfaction, more confidence in completion of deliverables, more perceived institutional resources for collaboration, better impressions, better interpersonal collaborations
Hall et al. [12]^b^	Cross-Disciplinary Collaboration Activities Scale: Perceived frequency of engagement in collaborative activities outside of one’s primary field		Please assess the frequency with which you typically engage in each of the activities listed below (e.g., read journals or publications outside of your primary field)	9	7-point Likert scale ranging from “never” to “weekly”	Cronbach’s alpha = 0.81	Convergent validity: Higher frequency of cross-disciplinary collaborative activities correlated with stronger multidisciplinary and interdisciplinary/transdisciplinary research orientation
Hanel and St-Pierre [55]	Originality of Innovation: Firm representatives’ perceptions/ ratings of most important innovation in terms of originality	5944 manufacturing provincial enterprises included in the Statistics Canada Survey of Innovation 1999 that reported research and development collaborative arrangements with universities to develop new or significantly improved products or manufacturing processes during the previous 3 years (1997–1999)	Firms asked if most important innovation was a “world first,” a “Canadian first,” or a “firm first”	NA	NA	NR	NR
Mâsse et al. [19]^a^	Methods Index: Perceptions of new methods created (in general), specifically development or refinement of methods for gathering data	216 research faculty, staff, and trainees from NCI TTURC	Average of seven items – each item rated in written survey by participants (research faculty, staff, and trainees)	7	4-point Likert scale ranging from “no progress” to “excellent progress”; also option of “does not apply”		Convergent validity: Correlations with satisfaction with collaboration, impact of collaboration, trust and respect, and transdisciplinary integration
	Science and Models Index: Perceptions of new science and models of tobacco use; to include understanding multiple determinants of the stages of nicotine addiction		Average of 17 items; each item rated in written survey by participants (research faculty, staff, and trainees)	17			
	Improved Interventions Index: Perceptions of improved interventions developed (in general – most items not specific to tobacco use); specifically progress in pharmacologic interventions		Average of 12 items; each item rated in written survey by participants (research faculty, staff, and trainees)	12			
Oetzel et al. [23]^c^	Partnership Synergy: Partner’s ability to develop goals, recognize challenges, respond to needs, and work togetherDomain: Intervention/Research	138 PIs/PDs and 312 academic or community partners from 294 CBPR projects with US federal funding in 2009	Each item rated in written survey by participants. Final score used in analysis was an average of five items	5	5-point Likert scale:1 = not at all; 2 = very little; 3 = somewhat; 4 = mostly; 5 = to a great extent	Cronbach’s alpha = 0.90	Construct validity: Confirmatory factor analysis. Convergent validity: Correlations with structural/individual dynamics scales, relational dynamics scales, and other outcome variables
	Systems and Capacity Changes: Partner Capacity Building. Develops the skills to benefit individual membersDomain: Outcomes		Each item rated in written survey by participants. Final score used in analysis was an average of three items	4		Cronbach’s alpha = 0.80	
	Systems and Capacity Changes: Agency Capacity Building. Develops the reputation and the skills of agencies involved in the partnership Domain: Outcomes		Each item rated in written survey by participants. Final score used in analysis was an average of three items	4		Cronbach’s alpha = 0.87	
	Systems and Capacity Changes. Changes in Power Relations: Degree to which power and capacity has been developed in the community membersDomain: Outcomes		Each item rated in written survey by participants. Final score used in analysis was an average of five items	5	5-point Likert scale:1 = strongly disagree; 2 = disagree; 3 = neither agree nor disagree; 4 = agree; 5 = strongly agree	Cronbach’s alpha = 0.81	
	Systems and Capacity Changes: Sustainability of Partnership/Project: Likelihood of the project and partnership continuing beyond the funding periodDomain: Outcomes		Each item rated in written survey by participants. Final score used in analysis was an average of three items	3		Cronbach’s alpha = 0.71	
	Health Outcomes: Community Transformation: Policy changes and community improvementDomain: Outcomes		Each item rated in written survey by participants. Final score used in analysis was an average of four items	7	5-point Likert scale:1 = not at all; 2 = very little; 3 = somewhat; 4 = mostly; 5 = to a great extent	Cronbach’s alpha = 0.79	
	Health Outcomes: Community Health Improvement. Improvement of health for the community as a result of the project		Single item rated in written survey by participants: Overall, how much did your research project improve the health of the community?	1	5-point Likert scale:1 = not at all; 2 = a little; 3 = somewhat; 4 = quite a bit; 5 = a lot	NA	
Philbin [35]	Satisfaction: Perception of collaborators’ satisfaction (as indicator of social knowledge sharing and improvement)	None – measure proposed based on literature review and interviews with 32 university and industry representatives involved in research collaborations	Satisfaction of students, academic staff, and industrial contacts involved in the collaboration (no specifics provided)	NA	NA	NA	NA
	Value of Technology Improvement Delivered: Company’s perception of value of technology improvements delivered (as indicator of technology sustainability of collaboration)		Survey of company representatives to measure the value of technology improvements delivered associated with the research collaboration (no specifics provided)				
	University and company staffs’ perceptions of Incorporation of knowledge developed into continuing professional development (as indicator of technology sustainability of collaboration)		Measurement of how knowledge developed is being incorporated into continuing professional development for both university and company staff involved (no specifics provided)				
	Perceptions of relevance of research to company’s business objectives (as indicator of projects and business knowledge sharing and improvement)		Measure of relevance of research to company’s business objectives (no specifics provided)				
	University and company’s perceptions of the percentage alignment of research to organizational strategies (as indicator of project and business sustainability of collaboration)		Percentage alignment of research to organizational strategy, for both university and company perspectives (no specifics provided)				
	Perceptions of the extent of personal relationships between company and university resulting from the collaboration (as indicator of social sustainability of collaboration)		Numerical measure for the extent of personal relationships between company and university resulting from the collaboration (no specifics provided)				
	Perceptions of the level of interactions between senior levels of the collaborators, especially at company board level and senior academic faculty level (as indicator of social sustainability of collaboration)		Level of interactions between senior levels of the collaborators, especially at company board level and senior academic faculty level (no specifics provided)				
	Perceptions of the level of influence by university faculty on company’s corporate strategy (as indicator of social sustainability of collaboration)		Level of influence by university faculty on company’s corporate strategy (no specifics provided)				
Trochim et al. [17]^d^	Methods Progress Scale: Perceptions of progress on development of methods in last 12 months (Intermediate term outcome)	216 research faculty, staff, and trainees from NCI TTURC	Self-report survey items administered annually	7	4-point Likert scale:1 = no progress; 2 = some progress; 3 = good progress; 4 = excellent progress	NR	NR
	Science and Models Scale: Perceptions of progress on development of science and models in last 12 months (Intermediate term outcome)			17			
	Progress on Development of Interventions Index: Perceptions of progress on development of new and Improved interventions in last 12 months (Intermediate term outcome)			12			
	Policy Impact Index: Perceptions of progress on policy outcomes in last 12 months (long-term outcome)			4	Yes/no		
	Translation to Practice Index: Perceptions of progress on translation into practice outcomes in last 12 months (long-term outcome)			9			
	Health Outcomes Impact Scale: Perceptions of optimism regarding positive health outcomes from center research within next 5 years (long-term outcome)			6	5-point Likert scale:1 = not at all optimistic; 2 = somewhat optimistic; 3 = moderately optimistic; 4 = very optimistic; 5 = extremely optimistic		
***Peer review perceptions of outcomes***							
Hall et al. [12]^e^	Written Products Protocol: Cross-disciplinary collaboration and productivity	21 center developmental project proposals from four NCI TREC centers	Unclear how final score calculated	37 item protocol used to evaluate proposals. Dimensions of cross-disciplinarity assessed: disciplines represented, levels of analysis, type of cross-disciplinary integration, scope of transdisciplinary integration, an overall assessment of general scope of each proposal	Items describing proposal with various response formats; one item – rate whether “unidisciplinary”, “multidisciplinary”, “interdisciplinary” or “transdisciplinary” proposal, two items re: transdisciplinary integration and scope of proposal using 10-point Likert scale ranging from “none” to “substantial”	Inter-rater reliability of *r* = 0.24–0.69. Highest inter-rater reliability was for experimental types (0.69), number of analytic levels (0.59), disciplines (0.59), and scope (0.52). Lowest inter-rater reliability was for type of cross-disciplinary integration (0.24)	Convergent validity: Higher number of disciplines in proposal, the broader its integrative score, larger its number of analytic level. The higher the type of disciplinarity, the broader its overall scope
Philbin [35]	Knowledge Improvement Index: Level of knowledge improvement (as indicator of technology knowledge sharing and improvement)	None – measure proposed based on literature review and interviews with 32 university and industry representatives involved in research collaborations	Independent numerical rating of the level of knowledge improvement (unit of analysis not specified; no specifics provided)	NA	NA	NA	NA
Trochim et al. [17]^d^	Peer review of progress on development of methods in last 12 months (intermediate term outcome)	216 research faculty, staff, and trainees from NCI TTURC	Peer review of Subproject Annual Progress Report Summaries from seven centers for progress on outcome; two randomly assigned reviewers for each	1	5-point scale; anchors not specified	>80% agreement by both raters with no more than 1-point difference. Also, both Kendall’s tau b and Spearman’s rho demonstrated positive and significant agreement	NR
	Peer review of progress on development of science and models in last 12 months (Intermediate term outcome)		Peer review of Subproject Annual Progress Report Summaries from seven centers for progress on outcome; two randomly assigned reviewers for each	1			
	Peer review of progress on development of Interventions in last 12 months (Intermediate term outcome)		Peer review of Subproject Annual Progress Report Summaries from seven centers for progress on outcome; two randomly assigned reviewers for each	1			
***Qualitative descriptions of outcomes***							
Armstrong and Jackson-Smith [56]	Improved interdisciplinary understanding for: individuals, research team, and university/ systematic (Integrative Capacity)	24 team members: 11 academic researchers; 6 non-academic team members (from related organizations and companies); 7 graduate students	30- to 60-minute semi-structured interviews with qualitative analysis of interview transcripts	NA			
	Team capacity to work in integrated manner, for: individuals, research team, and university/systematic (Integrative Capacity)		30- to 60-minute semi-structured interviews with qualitative analysis of interview transcripts				
	Development of research plan for: individuals, research team, and university /systematic (Integrative Capacity)		30- to 60-minute semi-structured interviews with qualitative analysis of interview transcripts				
Hager et al. [53]	Perceptions of impact of faculty development collaborative research fellowship	Six interprofessional faculty fellows (dentists, pharmacists, physicians)	Qualitative observations made and recorded after each seminar or learning session and analyzed for themes at end of fellowship				
Stokols et al. [25]	Transdisciplinary Conceptual Integration; e.g., a transdisciplinary economic model (to assess the costs of smoking), new research proposal development by transdisciplinary teams, new directions for transdisciplinary collaborations	NIH TTURC investigators at three centers	Descriptions provided by TTURC investigators through open-ended “periodic interviews” from 1999 to 2004				
Vogel et al. [57]	Perceived impacts of transdisciplinary team science	31 investigators, staff, and trainees from the NCI TREC centers	15 question in-depth semi-structured interviews (interview guide provided)				
***Healtd indicators/outcomes***							
Aguilar-Gaxiola et al. [58]	60 specific categories of community health indicators (e.g., life expectancy; preventable hospitalizations) within an organizing structure of nine determinants of health (e.g., general health status)	21 health indicator projects	Not provided in this article	NA	NA		

NA, not applicable; NR, not reported; NIH, National Institutes of Health; CTSA, Clinical and Translational Science Award; NSF, National Science Foundation; ISI, Institute for Scientific Information; DOE, US Department of Energy; SCI, Science Citation Index; CV, curriculum vitae; NCI, National Cancer Institute; IF, impact factor; TTURC, Transdisciplinary Tobacco Use Research Centers; NHMFL, National High Magnetic Field Laboratory;RTI, relative team impact; TREC, Transdisciplinary Research on Energetics and Cancer; PI, principal investigator; PD, project director; CBPR, community-based participatory research.

a Details obtained by cross-referencing article (TTURC Researcher Survey 2002) from https://cctst.uc.edu/sites/default/files/cis/survey-TTURC_research.pdf [44].

b Details obtained by cross-referencing article (TREC Baseline survey) from https://www.teamsciencetoolkit.cancer.gov/Public/TSResourceMeasure.aspx?tid=2%26rid=36 [42].

c Original instrument available at http://cpr.unm.edu/research-projects/cbpr-project/index.html--scroll to 2 (Quantitative Measures – “Key Informant” and “Community Engagement” survey instruments). Developmental work on measures from Oetzel et al. (2015) continues in an NIH NINR R01 (Wallerstein [PI], 2015–2020 “Engage for Equity” study (see http://cpr.unm.edu/research-projects/cbpr-project/cbpr-e2.html).

d Details obtained by cross-referencing article (TTURC Researcher Survey 2002) from https://cctst.uc.edu/sites/default/files/cis/survey-TTURC_research.pdf [44] and from Kane and Trochim [59].

e Details obtained by cross-referencing article (NCI TREC Written Products Protocol 2006-09-27) from https://www.teamsciencetoolkit.cancer.gov/Public/TSResourceMeasure.aspx?tid=2%26rid=646 [43].

Measures were broadly classified into one of the six different categories, reflected in Table 2: (1) counts or numerical representations of products (e.g., number of publications; 38 measures); (2) quality indicators of counted products (e.g., journal impact factor; 7 measures); (3) self-reported perceptions of outcomes (e.g., perceived productivity; 32 measures); (4) peer-reviewed perceptions of outcomes (e.g., progress on the development of interventions; 5 measures); (5) qualitative descriptions of outcomes (e.g., descriptive data collected by interview; 6 measures); and (6) health indicators/outcomes (e.g., life expectancy; 1 overall measure with 60 different indicators). The number of items per measure ranged from a single count to a 99-item scale, with over 50% of the measures composed of a single count, number, or rating of a single item.

Twenty-three of the 89 measures were recommendations on measures and had no reported reliability or validity as would be expected [35]. For the remaining 66 measures, only 16 reported assessments of reliability. Nine of 24 measures in the self-reported perceptions category included Cronbach’s alpha >0.70, showing internal consistency reliability. Six measures (3 of 24 in the counts of products category and 3 of 4 in the peer-reviewed category) had inter-rater agreement described; all were over 80%. One measure in the peer-reviewed category reported inter-rater reliability of *r* = 0.24–0.69. Of these 16 measures with reported reliability, nine had some form of validity described: confirmatory factor analysis (6 measures) and convergent validity (3 measures). Of the remaining 50 measures without reliability data, five had some type of convergent validity described and one was supported by principal component analysis. Once again, convergent validity was not formally labeled as such but was evident in terms of correlations between the measure under study and other relevant variables.

## Discussion

### Quality Measures

Overall, there are a relatively large number of scales, some of them robust, that have been used to measure the quality or process of research collaborations (e.g., trust, frequency of collaboration). However, many scales have not been extensively used and have been subjected to relatively little repeated psychometric study and analysis. Most have been developed in support of a particular research project rather than with the intent of becoming a standard indicator or scale for the field. Although calculated across multiple organizations, estimates of reliability and/or validity were often study specific as well. Reports of effect sizes (sensitivity or responsiveness) were rare and limited to correlations, and construct validity has not been explored beyond exploratory or confirmatory factor analyses. Given this dearth of replicated psychometric data, it is not surprising that widely accepted, standard scales have not emerged to date. Wide-scale testing of measures of collaboration is essential to establish reliability, validity, and sensitivity or responsiveness across settings and samples.

Scales developed to date have been primarily focused on group dynamics (including the quality of interpersonal interactions, trust, and communication). Although these are important factors, few measurements have been made of how well a team functions (such as leadership styles) and the degree to which the team’s work is viewed as synergistic, integrative, or otherwise more valuable than would occur in a more siloed setting. Oetzel et al.’s [23] beginning psychometric work provides an example of some of these types of measures. This is in contrast to the numerous available (or under development) scales to measure attitudes toward collaborations and quality of collaborations that exist at specific institutions.

Despite these limitations, two sets of measures deserve note. First, those reported by Hall et al. [12] and Mâsse et al. [19] as measures of collaborations in National Cancer Institute-funded Transdisciplinary Tobacco Use Research Centers have been used more extensively than many of the other scales in this review as indicators of collaboration quality among academic partners (although relatively little additional psychometric data have been reported beyond initial publications). Second, the measures reported by Oetzel et al. [23] are unique in that they are scales to assess research quality involving collaborations between academics and communities, agencies, and/or community-based organizations. They are also unique in representing responses from over 200 research partnerships across the USA. This review did not distinguish between partnerships (e.g., involving just two partnering organizations) and coalitions (involving multiple organizations).

### Outcome Measures

Similar to measures of collaboration quality, little agreement exists as to how to best measure outcomes of research collaborations. By far, the most common type of measurement is a simple count of products over a set period of time (e.g., publications, grants, and/or patents). Interestingly, the procedures used for counting or calculating these products are rarely reported and therefore are not replicable. In addition, published reports infrequently include any type of verification of counts, leaving the reliability of such counts or calculations in question.

The second most common type of measure is the use of self-reported scales to quantify the researchers’ perceptions of collaboration outcomes. These include measures of perceived productivity or progress, changes in relationships with partners, increased capacity, and sustainability. Few of these measures, with the exception of the psychometric works of Hall et al. [12] and Oetzel et al. [23], have documented reliability and validity. In general, despite a relatively large number of scales, most of these were not developed for the purpose of becoming standard indicators or measures and most have had little psychometric study or replication.

Efforts to measure the quality of counted products, such as consideration of citation percentiles, journal impact factors, or field performance indicators, offer important alternatives in the quantity versus quality debate and actually may be useful for evaluating the long-term scientific impact of collaborative outcomes. Likewise, peer-reviewed ratings of outcomes based on reviews of proposals or progress reports could provide more neutral and standardized measures of collaboration impact. Both of these categories of measures are used infrequently but could have significant influence if applied more widely in the evaluation of collaborative work. However, further work on a reliable rating’s scale for use in peer review is needed before it is able to provide comparable results across studies.

### Recommendations

Remarkably, the results of this review, which defines research collaborations to include different types of collaborative partnerships, are very similar to reviews of measures of community coalitions [60] and community-based participatory research [61] conducted 15 and 7 years ago, respectively. Both of those studies concluded that there are few reliable and valid measures. In the intervening years, some progress has been made as noted [see Refs. 12, 19, 23 as examples]. Based on this observation and our findings in this study, we offer six recommendations to advance the field of team science: (1) We must pay careful attention and devote resources to the development and psychometric testing of measures of research collaboration quality and outcomes that can be replicated and broadly applied. Measures listed in this review with solid initial reliability and validity indicators provide reasonable starting points for continued development; however, measures of other constructs will also be necessary. (2) To establish validity for use in different populations and settings, designed measures should be tested across various research partner and stakeholder relationships (e.g., academia, industry, government, patient, community, and advocacy groups). (3) When evaluating outcomes, it is critical that we focus on both the quality and quantity of products and the use of rating scales for peer review. (4) The sensitivity and responsiveness of measures to interventions should be evaluated as an additional psychometric property. (5) Publications reporting on assessments of collaborations should include a clear description of the measures used; the reliability, validity, and sensitivity or responsiveness of the measures; and a statement on their generalizability. (6) Reports incorporating the use of narrowly applicable measures should include a justification for not using a more broadly applicable measure.

## Conclusions

Although a few studies have conducted exemplary psychometric analyses of some measures of both collaboration quality and outcomes, most existing measures are not well-defined; do not have well-documented reliability, validity, or sensitivity or responsiveness (quality measures); and have not been replicated. Construct validity, in particular, requires further exploration. Most of the reported measures were developed for a single project and were not tested across projects or types of teams. Published articles do not use consistent measures and often do not provide operational definitions of the measures that were used. As a result of all of these factors, it is difficult to compare the characteristics and impact of research collaborations across studies.

Team science and the study of research collaborations are becoming better and more rigorous fields of inquiry; however, to truly understand the reasons that some teams succeed and others fail, and to develop effective interventions to facilitate team effectiveness, accurate and precise measurements of the characteristics and the outcomes of the collaborations are needed to further translational science and the concomitant improvements in public health.
